# The Effect of Toileting Position in Uroflow Curves in Young Healthy Nulliparous Women

**DOI:** 10.1155/2019/5273083

**Published:** 2019-11-03

**Authors:** Omar Felipe Dueñas-Garcia, Maria del Pilar Matta-Gonzalez, Kylie Fuller, Wei Fang, Robert Edward Shapiro

**Affiliations:** Obstetrics and Gynecology Department, West Virginia University, Morgantown, West Virginia, USA

## Abstract

The objective of our study was to determine the effect of voiding positions on uroflow parameters in young, healthy nulliparous women with no pelvic floor disorders. *Material and Methods*. From December 2017 to February 2018, we performed a single-institution cross-sectional study with 30 healthy volunteers comparing uroflow curves in sitting and hovering positions. 49 participants were initially prescreened with a validated tool questionnaire for pelvic floor disorders and 30 participants who had absent symptoms were included for the final analysis. From the selected participants, demographics were collected and comparisons between the sitting and hovering position groups regarding the maximum flow rate (Qmax), average flow rate (Qave), voided volume (VV), and time to peak flow (TQmax) were conducted using either the paired *t*-test or the Wilcoxon rank sum test. In addition, linear regression analysis was performed to determine whether height, BMI, and age have significant impact on the log-transformed average of the pre- and postvalues of either Qmax, Qave, VV, or TQmax, as the average of these values are not normally distributed. *Results*. There were no statistical differences between the hovering and sitting position groups on the maximum flow rate (*p*=0.93), average flow rate (*p*=0.82), voided volume (*p*=0.53), and time to peak flow (*p*=0.82). BMI had borderline significant impact on Qave with *p* value = 0.0531. *Conclusion*. Different voiding toileting habits do not affect the most commonly used uroflow parameters in young healthy nulliparous patients. Results need to be corroborated by a larger scale study considering the small sample size of our study.

## 1. Introduction

Uroflow studies are a noninvasive test to assess voiding function in many urology and urogynecology practices. Uroflowmetry can be used when patients report voiding symptoms, defined as a departure from the normal sensation or function experienced by women during or following the act of micturition. Uroflow measurements and postvoid residual (PVR) volumes influence the operative plan in women undergoing surgery for pelvic organ prolapse and/or urinary incontinence [1].

Largely because of cultural hygiene practices, women adopt different positions when voiding in public restrooms. It is not uncommon to describe that some patients do not sit on the toilet because of fear of contamination. A prior study from 1991 reported that up to 85% of the subjects attending a gynecology clinic hovered or crouched on the seat of the public toilets [2].

The effect of voiding positions and uroflow parameters on the voiding function has not been established. Our primary objective was to determine the effect on uroflow parameters of two voiding positions, i.e., hovering and sitting on voiding function in a healthy nulliparous prescreened population with absence of pelvic floor disorder symptoms.

## 2. Materials and Methods

From December 2017 to February 2018, we performed a single-institution cross-sectional study with 30 healthy volunteers comparing uroflow curves in the sitting and hovering position. The Internal Review Board of West Virginia University approved the study (Morgantown, WV). It was investigator initiated and departmental funds were used. Additional support for the data analysis was obtained through the West Virginia University Clinical Trials Institute (WVUCTSI), National Institute of General Medical Sciences of the National Institutes of Health under Award Number 2U54GM104942-02. The ICIQ questionnaires were facilitated by the Bristol Urologic Institute which allowed us to use the instruments for this research project.

### 2.1. Study Population

30 participants were healthy young females recruited through our clinic and flyers posted at our institution. We defined healthy as those subjects who were nulliparous, nonpregnant, 18 years old or older, not being pregnant, not taking medications, no history of urological or gynecological procedures, and had a score of “0” at the International Continence Questionnaire Bristol Female Lower Urinary Tract Symptoms Questionnaire, (ICIQ-BFLUT-SF), the International Continence Questionnaire—Vaginal Symptoms (ICIQ-VS), and the International Continence Questionnaire—Urinary Incontinence (ICIQ-UI) (ICIQ-UI) questionnaires. Volunteers were compensated for their time with a $20 coffee gift card.

### 2.2. Study Measurement of the Outcome

Uroflow measurements were performed with a “UROCAP IV LIGHT Wireless uroflow meter” from Laborie Mississauga, ON, Canada. The uroflow machine was set in a private room designated for urodynamic testing at our clinic. Subjects voided when they felt that reached capacity in a private urodynamic room with a lock door. Subjects were instructed to void in the squatting and sitting position at the uroflow. To illustrate the positions, we used the Wang's validated questionnaire [3].

After voiding in one position, subjects had the option to return in a different day or time under the same conditions except for a different position.

### 2.3. Study Outcomes

Those 30 Volunteer data were collected using Research Electronic Data Capture (RedCap), a web-based safe collection tool. Demographics and subject's characteristics were described with frequency and percentages. Comparison of the maximum flow rate (Qmax), Average flow rate (Qave), voided volume (VV), and time to peak flow (TQmax) between the two positions were conducted using either the paired *t*-test or the Wilcoxon signed ranks test.

A linear regression analysis was conducted to evaluate the impact of the volunteer's characteristics such as BMI, age, and height on urodynamic parameters, defined as the average of pre- and post- Qmax, Qave, VV, and TQmax.

The data analysis was performed using SAS (Version 9.4).

## 3. Results

Forty-nine subjects were prescreened for the study, and we only included 30 for the final analysis. Subjects were excluded due to taking medications (2), having a medical condition (2), ICIQ questionnaires with a score ≥1 (14), or because of their age (1). For the patient characteristics, the mean age was 24.9 (18–35– years old) years, had an average height of 1.61 mts (1.52–1.78 mts), an average BMI of 25.65 (18.3–30.4), and average weight of 67.03 (45–86) kg. For the race and ethnicity, 26 were self-identified whites, 2 Hispanics, 1 African-American, and 1 Asian.

In Table 1, we noticed that there were no significant differences between the uroflow parameters Qmax, Qave, VV, and TQmax voiding in the sitting and hovering position.

Table 2 shows the uroflow parameters in the sitting position alone. There was no significant change in percentile range from changing voiding posture. We then decided to compare the mean values of the uroflow parameters of interest with what is considered to be as the established average (Table 3). Datasets from previous studies were not available and no statistical comparisons with our data were made.

From the linear regression analysis, height, BMI, and age basically did not have significant impact on log-transformed average of the pre- and postvalues of either Qmax, Qave, VV, or TQmax. The only exception was the positive association between peak flow with BMI and age, showing that the older the subject and the higher the BMI, the faster the time to peak flow.

## 4. Discussion

We found that the voiding position does not influence basic urodynamic parameters within young, healthy nulliparous women.

Prior studies have shown conflicting evidence about our findings. A study performed in the UK showed a reduced Qave and Qmax in the crouching position as well as elevations in the postvoid residual. A Turkish and a Taiwanese study found no differences between the different voiding positions.

Previous studies have mixed populations including men and women [5–7], or they included patients visiting the gynecology clinic like the British study, where the mean age of the subjects was 47.8 years [2].

Unfortunately, none of the previous studies actually prescreened their volunteers with a validated questionnaire for the absence of pelvic floor disorders, including urinary incontinence or lower urinary tract symptoms. As an interesting finding in our study, 14 subjects who were self-defined as healthy and nulliparous had either lower urinary tract, urinary incontinence, and/or vaginal symptoms. This highlights the relevance of either doing an exam or using a validated tool to determine the absence of pelvic floor symptoms, and this may explain why previous similar studies found contradictory findings.

In this study, we used the ICIQ-BFLUT-SF, ICIQ-VS, and ICIQ-UI questionnaires. These instruments have been extensively validated, and they show excellent reliability screening populations for vaginal symptoms (ICIQ-VS), lower urinary tract symptoms (ICIQ-LUTS), and urinary incontinence (ICIQ-UI) [8–10]. Specifically, in the case of the questionnaire for lower urinary tract symptoms, the questionnaire is specific for females [9].

In our study, from the studied variables, the BMI and the age had a positive association with the peak flow. The significance of this is unknown, but we consider that it is also possible that this is a confounder and the BMI and age are also correlated. The BMI and fast peak flow can be explained by increasing the intrabdominal pressure during voiding, but more studies are required to prove this hypothesis. Another possible explanation for these findings may be that with increasing age and BMI, more motor units are needed to maintain continence with resultant increased structural integrity of the urethral sphincter. This is consistent with a well-established hypothesis where larger and more motor units are recruited with greater muscle contraction [11].

Height was shown to be inversely proportional to peak flow, meaning the taller the patient the longer the voiding time. The *p* value is also >0.05 The researchers hypothesize that uroflow parameters in taller individuals may reflect a slightly overall longer urethral length which could explain this result. To the best of our knowledge, this finding has not been replicated in other studies. Pomian et al. reported a wide variation in the urethral length ranging from 19 to 45 mm with BMI being associated with a longer urethra but not patient height [12].

One promising finding is that BMI, with *p* value = 0.0531 and negative relationship with Qave, had borderline significance on Qave. But, our study was not powered to detect a difference on that variable and further studies should be conducted to determine if there is a true association.

Limitations of the study include a small sample size that was not ethnically or racially diverse. Although adequately powered, budget and resource limitations prevented us from obtaining more subject volunteers that were more representative of the broader population. We acknowledge that toileting behaviors can vary substantially based on different geographic regions. Gupta et al. reported a Qmax of 18.4 + −.2 ml/s from Indian women in the sitting position, whereas Unsal et al. reported a Qmax of 28.09 + 0.66 ml/s in their study of Turkish women in the same position [5].

In some regions of the world, toileting positions can include squatting. We did not include this toileting position as part of our study since this is not a common voiding position in the United States. However, prior research has been done with squatting and has demonstrated conflicting results. Rane and Corstianns showed no statistically significant differences in uroflow parameters with squatting [13]. Gupta et al. showed the squatting posture was associated with a significantly higher maximum flow rate and lower postvoid residual [14]. Further research should include squatting as a toileting position to gain further perspective on the voiding function.

The study participants were not directly observed while voiding may also be considered another limitation of the study. However, many people are known to have urinary difficulty in situations where others are in proximity. Because direct observation could cause voiding difficulty and ultimately influence uroflow results, we felt it best to allow our study participants to void in a private setting. Of note, most of the volunteers were medical students, relatives of them, or resident physicians. Thus, we believe they provided a truthful performance during the execution of the intervention.

## 5. Conclusion

Different toileting positions do not affect the most commonly used uroflow parameters in young, healthy nulliparous subject volunteers. As age, BMI, and height do not have a significant correlation with uroflow parameters, they should not be taken into consideration when prescribing treatments. Results from this study can be considered as preliminary, considering the small sample size and nondeterminant results. Further studies are required to corroborate our findings.

## Figures and Tables

**Table 1:** Comparison of uroflow parameter values from the same patients sitting and hovering using hypothesis testing test.

Parameters	Mean sitting	Mean hovering	*p* values
Maximum flow rate (Qmax)	28.5 mL/s	27.5 mL/s	0.9329
Average flow rate (Qave)	15.65 mL/s	16.4 mL/s	0.8154
Time to peak flow (TQmax)	6.35 s	5.75 s	0.8176
Flow time	15.2 s	16.4 s	0.955
Voided volume (VV)	322 mL	294 mL	0.9199

**Table 2:** Uroflow parameter values in the sitting position.

Parameters	Minimum	10 perc	25 perc	50 perc	75 perc	90 perc	Maximum
Maximum flow rate (Qmax)	13 mL/s	16 mL/s	18.75 mL/s	28.5 mL/s	38.75 mL/s	47.9 mL/s	58 mL/s
Average flow rate (Qave)	7.5 mL/s	9.03 mL/s	12.05 mL/s	15.65 mL/s	23.675 mL/s	27.79 mL/s	31.8 mL/s
Time to peak flow (TQmax)	1.4 s	2.8 s	3.95 s	6.35 s	8.55 s	10.94 s	24.8 s
Flow time	5.5 s	7.29 s	9.475 s	15.2 s	24.9 s	36.21 s	47.6 s
Voided volume (VV)	57 mL	94 mL	119 mL	322 mL	484 mL	654.5 mL	864 mL

**Table 3:** Comparison of uroflow parameter values in the sitting position to the normal uroflow parameters established in the literature.

Parameters	Experimental average	Established average^*∗*^
Maximum flow rate (Qmax)	29.7 mL/s	34.8 mL/s
Average flow rate (Qave)	19.97 mL/s	15.4 mL/s
Time to peak flow (TQmax)	6.98 s	7.8 s
Flow time	18.43 s	26.5 s
Voided volume (VV)	333.07 mL	409.2 mL

^*∗*^Data obtained from the review article of “Normal urodynamic parameters in women” by Mahfouz, et al. [4].

## Data Availability

The data used to support the findings of this study are available from the corresponding author upon request.
